# Acute Creatine Ingestion Before Resistance Training Enhances Strength Performance More than Ingestion During or After Training: A Randomized Crossover Pilot Trial

**DOI:** 10.3390/nu18111789

**Published:** 2026-06-01

**Authors:** Khouloud Ben Maaoui, Slaheddine Delleli, Arwa Jebabli, Nourhène Mahdi, Juan Del Coso, Hamdi Chtourou, Luca Paolo Ardigò, Ibrahim Ouergui

**Affiliations:** 1 High Institute of Sport and Physical Education of Sfax, University of Sfax, Sfax 3000, Tunisia; benmaaouikhouloud88@gmail.com (K.B.M.); sdelleli2018@gmail.com (S.D.); jebabliarwa@gmail.com (A.J.); nourhene648@gmail.com (N.M.); h_chtourou@yahoo.fr (H.C.); 2 Research Laboratory: Education, Motricity, Sport and Health, EM2S, LR19JS01, University of Sfax, Sfax 3000, Tunisia; 3 Physical Activity, Sport and Health Research Unit, UR18JS01, National Sport Observatory, Tunis 1003, Tunisia; 4 Sport Sciences Research Centre, Rey Juan Carlos University, 28943 Fuenlabrada, Spain; juan.delcoso@urjc.es; 5 Department of Teacher Education, NLA University College, 0166 Oslo, Norway; 6 High Institute of Sport and Physical Education of Kef, University of Jendouba, Kef 7100, Tunisia; 7 Research Unit: Sport Sciences, Health and Movement, UR22JS01, University of Jendouba, Kef 7100, Tunisia

**Keywords:** strength performance, ergogenic aid, nutrient timing, dietary supplement, resistance training

## Abstract

**Background/Objectives**: Although creatine (Cr) supplementation is well established for enhancing strength exercise adaptations, limited evidence exists regarding whether the timing of a single Cr dose relative to exercise acutely influences performance and related physiological and perceptual responses. This study examined whether the timing of a single dose of Cr ingestion relative to a strength exercise session influences acute strength and power performance, cognitive function, perceptual responses, and selected blood biomarkers in physically active men. **Methods**: In a randomized, placebo-controlled crossover design, 11 physically active men (26.09 ± 4.39 years) completed five experimental conditions: Cr ingested before exercise (CrB), during exercise (CrD), and after exercise (CrF), placebo (PL), and a no-supplement control. Participants ingested 0.1 g·kg^−1^ body mass of monohydrate Cr or placebo. Each condition included a standardized strength training session, where bench press (BP) and back squat (BSQ) performance was assessed as the total external load lifted (kg) across six sets performed at 80% of 1-RM for each exercise. Countermovement jump (CMJ) performance, Profile of Mood States (POMS), cognitive performance (digit cancelation test), perceived exertion (RPE), perceived recovery scale (PRS), Delayed-Onset Muscle Soreness (DOMS), and blood markers of muscle damage and renal function were assessed after the resistance training session. Data were analyzed using repeated-measures ANOVA or non-parametric equivalents, with post hoc comparisons adjusted for multiple testing. **Results**: There was a significant main effect of condition for both BP (F = 4.91, ηp^2^ = 0.33, *p* = 0.035) and BSQ performance (F = 33.22, ηp^2^ = 0.77, *p* < 0.001), with greater performance under the CrB condition compared with PL and control (*p* < 0.05). A significant effect of condition was also observed for creatine kinase (χ^2^ (4) = 12.22, *p* = 0.016) and creatinine concentrations (χ^2^ (4) = 17.75, *p* = 0.001). Blood creatine kinase concentrations were greater under CrF conditions than control (*p* = 0.013) and PL (*p* = 0.041). Moreover, creatinine concentration was lower under the CrB condition compared to CrD (*p* = 0.033), CrF (*p* = 0.003), and the control (*p* = 0.021). No differences were observed for CMJ performance, cognitive performance, POMS, RPE, PRS, DOMS, or the remaining biochemical markers across treatments. **Conclusions**: Pre-exercise creatine ingestion (without loading phase) was associated with greater acute strength performance compared with other timing conditions. However, the findings are exploratory and have to be confirmed with a higher sample size and robust placebo/control structures.

## 1. Introduction

Creatine (Cr) (α-methyl guanidino-acetic acid) is primarily synthesized endogenously in the kidneys and liver [1,2]. However, Cr can be supplemented exogenously by taking commercially available Cr preparations, with creatine monohydrate being the most common form [3]. Specifically, it is well-established that Cr intake enhances intramuscular Cr reserves, muscle morphology (e.g., lean body mass), and functional performance (e.g., strength, endurance) [3,4,5]. Cr supplementation regimens typically consist of a loading phase of 20 g daily for 5–7 days, followed by a maintenance phase of 3–5 g daily to maintain high intramuscular Cr concentrations [6]. The initial loading phase aims to rapidly increase Cr reserves, while the subsequent maintenance dose aims to prevent Cr depletion [7].

The timing of Cr supplementation in relation to exercise has been hailed as a crucial factor in maximizing training gains [8]. The importance of the timing of Cr administration is based on the assumption that exercise-induced physiological changes, such as increased hyperemia and altered transport dynamics, may optimize tissue absorption and storage [9]. Yet, evidence supporting specific timing strategies is restricted to a few chronic studies with conflicting findings [10,11,12]. For instance, Antonio and Ciccone [10] reported superior gains in fat-free mass and strength with post-exercise versus pre-exercise Cr ingestion over a 4-week resistance program. However, their findings are constrained by the lack of a placebo group, small sample size, and reliance on magnitude-based inferences. Conversely, Candow et al. [11] found distinct differences between pre- and post-exercise ingestion regarding strength and hypertrophy over a 32-week intervention in older adults. More recently, Mills, Candow, Forbes, Neary, Ormsbee and Antonio [12] demonstrated that intra-training Cr supplementation enhanced strength and endurance compared to a placebo, but the lack of traditional pre- or post-exercise comparator groups precludes definitive conclusions about intra-workout efficacy.

Although resistance exercise is known to stimulate muscle Cr accumulation [13,14], current evidence regarding timing efficacy is entirely limited to chronic interventions with two-point comparisons being considered. Therefore, this study examines whether the timing of a single dose of Cr ingestion relative to a resistance training session influences acute strength and power performance, cognitive function, perceptual responses, and selected blood biomarkers in physically active men. Because pre-exercise ingestion ensures peak plasma Cr concentrations coincide with exercise-induced skeletal blood flow [8], it was hypothesized that pre-exercise Cr supplementation would yield the greatest enhancements in subsequent performance and cognitive metrics.

## 2. Materials and Methods

### 2.1. Participants

The sample size required for this study was estimated a priori using the G*Power software (version 3.1.9.4, University of Kiel, Kiel, Germany). Based on a crossover study with a repeated-measures ANOVA design with five conditions, α = 0.05 and power = 0.80, a minimum of 10 participants was estimated to be sufficient to detect moderate effects of the conditions (effect size f = 0.39), based on previous study [14] about the timing of Cr ingestion around resistance exercise performance on a bench press (BP), to reach an actual power of 84%. To account for potential attrition, 11 physically active men (mean ± SD; age: 26.09 ± 4.39 years; height: 176.36 ± 7.24 cm; body mass: 83.20 ± 9.79 kg) were recruited to participate in the present study. Participants were eligible for inclusion if they met the following criteria: (1) were non-smokers and did not consume alcohol or use anti-inflammatory medications during the experimental period or for at least one month before study commencement; (2) were free from musculoskeletal injury; and (3) demonstrated stable recovery status, defined as Hooper Index subscale scores ≤ 4 or a total score ≤ 16 for three consecutive days, in accordance with practical recommendations used in applied sport science settings [15,16]. Participants were excluded if they were taking medications, had consumed creatine monohydrate or creatine-containing dietary supplements within four weeks before the start of the study, followed a vegetarian diet, or had pre-existing renal or hepatic disorders. All participants received a detailed explanation of the study procedures, including potential risks and benefits, and provided written informed consent before participation. The study was conducted in accordance with the Helsinki Declaration, and the protocol was completely approved by the local Research Ethics Committee (CPP: N° 0414/2022) (approval date: 26 August 2022).

### 2.2. Experimental Design

This was a pilot, randomized, placebo-controlled crossover trial that followed the Consolidated Standards of Reporting Trials (CONSORT) guidelines for randomized crossover trials [17] (Supplementary Table S1) after being prospectively registered in the Pain Africa Clinical Trials Registry (PACTR202309597156293, 18 September 2023). The study was designed to examine the acute effects of Cr supplementation administered at different time points relative to exercise onset in conjunction with a strength training session. The primary outcome was strength performance, assessed as the total external load lifted during the bench press and back squat exercises. Secondary and exploratory outcomes included countermovement jump performance, cognitive function, perceptual responses, mood state, and selected blood biomarkers.

A familiarization session was conducted one week before the testing sessions. During this session, participants were familiarized with all testing procedures and exercise techniques used in the study to minimize potential learning effects. On the same day, participants’ anthropometric characteristics were determined, the relative dose for each participant was calculated, and the one-repetition maximum strength (1-RM) for the BP and back squat (BSQ) was established. The 1-RM for both BP and BSQ was determined using McGuigan’s methodology [18]. Briefly, participants completed a warm-up set of 4 repetitions at 50% of their predicted 1-RM. Participants performed successive 1-RM lifts starting with about 70% of the anticipated 1-RM and increased by 5–10 kg until their 1-RM was reached. There was a two-minute rest interval between sets [10]. The repetition with the highest load that was executed safely without assistance was regarded as the 1-RM. Throughout the experimental sessions, participants were tested under five conditions, including four supplementation conditions and one no-supplement control condition. In the supplementation sessions, participants ingested either creatine monohydrate (Eric Faver’s Nutrition Expert Creatine Pro Zero; EU, France, Villecheneuve and Puteaux) or a placebo at a dose of 0.1 g·kg^−1^ body mass, diluted in 500 mL of water. Cr supplementation was administered at three different time points relative to the strength training session: (1) before exercise (CrB), in which the full Cr dose was consumed as a single bolus 2 h before training; (2) during exercise (CrD), in which the total Cr dose was evenly distributed across the training session and consumed in equal aliquots between sets; and (3) after exercise (CrF), in which the full Cr dose was consumed immediately following the training session. In the placebo condition (PL), participants ingested an isovolumetric placebo solution (cornstarch maltodextrin, 0.1 g·kg^−1^ body mass) following the same ingestion timing as the pre-exercise condition (single bolus consumed 2 h before training). To avoid identification and ensure blinding success, supplements were administered in opaque, unmarked containers and handled by an independent person. In the control condition, no substance was ingested. For each experimental condition, participants completed an identical, standardized strength exercise session. The session consisted of two exercises performed in the following order: bench press and then back squat. Each exercise comprised six sets performed at 80% of the participant’s predetermined 1-RM, with repetitions decreasing across sets (12, 11, 10, 9, 8, and 7 repetitions), based on a previous study [19]. Inter-set rest intervals were fixed at 2 min, and a 3–4 min rest period was provided between exercises. All sessions were supervised by the same investigators to ensure proper technique and adherence to the prescribed protocol.

Participants were randomized to the order of experimental conditions using a structured block-randomization procedure with rank-based allocation to ensure balanced condition sequences across participants and to minimize potential allocation and order bias. Specifically, a random number was assigned to each participant using the RAND () function in Microsoft Excel 2007 (Redmond, WA, USA). Participants were then ranked in ascending order based on these random numbers. The treatment conditions were allocated according to rank. Specifically, participants were assigned to control, PL, CrB, CrD, and CrF, ordered by ascending rank, with a block size of three for the control arm and blocks of two participants for the other arms. An acute Cr dosage of 0.1 g·kg^−1^ was chosen since it showed its effectiveness in improving muscle performance when paired with strength exercise [11]. During exercise, performance was assessed as the total external load lifted (kg) across all sets for the bench press and back squat exercises performed at 80% of each participant’s predetermined 1-RM. To evaluate physical performance, participants completed a countermovement jump (CMJ) test to assess lower-body power [20] as well as repeated bench press (BP) [21] and back squat (BSQ) [22] tests to assess strength performance. These assessments were performed after completion of the training session. The BP and BSQ tests were conducted using a Smith machine (Atlantis, Laval, QC, Canada), and in the present study, both tests demonstrated good-to-excellent test–retest reliability, with intraclass correlation coefficients (ICCs) of 0.86 and 0.98, respectively. CMJ performance was assessed using a Myotest Pro accelerometer system (Myotest SA, Sion, Switzerland), which also demonstrated excellent test–retest reliability in the present study, with an ICC of 0.97.

Immediately after testing, venous blood samples (10 mL) were collected from an antecubital vein into heparinized tubes for the assessment of muscle damage markers (creatine kinase [CK], lactate dehydrogenase [LDH], aspartate aminotransferase [AST], alanine aminotransferase [ALT], and alkaline phosphatase [ALP]), renal function (creatinine), and electrolyte concentrations (Na^+^ and K^+^). Samples were placed in an ice bath (30 min of collection) and centrifuged immediately at 2500 rpm (× *g*) for 10 min. To avoid variations in assay conditions, all samples were analyzed in the same assay run. CK and LDH were determined using the 340 nm kinetics method. The blood samples were analyzed with the ELITechGroup kit (Puteaux, France).

**Figure 1:**
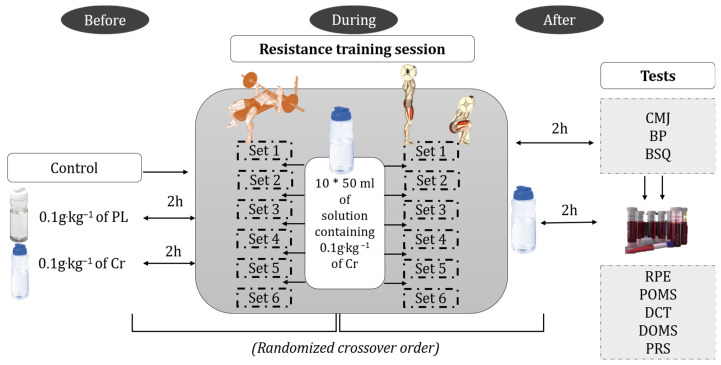
Schematic representation of the study. CMJ: countermovement jump; BP: bench press; BSQ: back squat; RPE: rate of perceived exertion; POMS: Profile of Mood States; DCT: digit cancelation test; DOMS: delayed-onset muscle soreness; PRS: perceived recovery scale; Cr: creatine; PL: placebo.

Following blood sample collection, participants completed subjective and cognitive assessments, including ratings of perceived exertion (RPEs) using the Borg scale [23], mood state evaluation using the Profile of Mood States (POMS [24]), and cognitive performance assessment using the digit cancelation test (DCT) [25]. Five minutes after completion of these assessments, participants reported their perceived recovery status using the perceived recovery scale (PRS; [26]) and their delayed-onset muscle soreness using a visual analog scale for Delayed Muscle Soreness (DOMS; [27]) (Figure 1). A washout period of 7 days was given between conditions to ensure sufficient recovery between conditions and eliminate the effects of the single dose of Cr ingested [28]. Such a period was suggested to be sufficient to eliminate Cr residuals after a single dose [28,29]. During the experimental period, participants were asked to maintain their habitual diet and sleep hygiene. To avoid the effect of circadian rhythm, all testing sessions were conducted at the same time of day (from 18h00 to 21h00).

### 2.3. Statistical Analyses

The statistical analysis was performed using SPSS software version 27.0.1. Data are presented as means ± standard deviation for normally distributed variables, and as medians with interquartile ranges for variables with non-normal distributions. The Shapiro–Wilk test was used to assess the normality of data distributions, and Levene’s test was used to verify homogeneity of variances. Sphericity was assessed using Mauchly’s test, and the Greenhouse–Geisser correction was applied when the assumption of sphericity was violated, as occurred for BP and BSQ. Strength performance, assessed as total external load lifted during the bench press (BP) and back squat (BSQ), was defined as the primary outcome of the study. All other outcomes, including CMJ performance, biochemical markers, mood state, cognitive performance, RPE, PRS, and DOMS, were considered secondary or exploratory outcomes. For normally distributed variables, including BP, BSQ, ALP, K^+^, Na^+^, vigor, DCT, and DOMS, a one-way repeated-measures analysis of variance (ANOVA) was used to examine the effect of the conditions. When a significant main effect was detected, Bonferroni-adjusted post hoc comparisons were performed to identify pairwise differences between conditions. Effect sizes were calculated as partial eta-squared (ηp^2^). The effect size statistic (ηp^2^) was calculated to assess the magnitude of difference using the following criteria: 0.01, 0.06, and 0,14 represent small, moderate, and large effect sizes, respectively [30]. For non-normally distributed variables (i.e., CMJ, CK, LDH, ALT, AST, creatinine, fatigue, anger, depression, tension, confusion, RPE, PRS), the Friedman test was used with the Wilcoxon signed-rank test for post hoc analysis. To assess the magnitude of the differences, the rank biserial correlation coefficient (r) was calculated using the Wilcoxon Z-scores and the total number of observations (N) (i.e., r = Z/√N), with results categorized as 0.1 to <0.3 (small), 0.3 to <0.5 (moderate), and ≥0.5 (large) [31]. The level of statistical significance was set at *p* < 0.050. To reduce the risk of Type I error, a hierarchical approach was adopted with strength performance (i.e., BP and BSQ) defined as the primary endpoint, while the power performance, physiological, and psychological variables were treated as secondary/exploratory outcomes.

Post hoc statistical power was also calculated for the main outcomes and is reported in Supplementary Table S2. These calculations were performed using G*Power software based on the observed effect sizes, sample size, number of repeated conditions, α = 0.05, and the statistical model used for each outcome. Because post hoc power estimates are dependent on the observed effects and may be unstable in small samples, these analyses were considered descriptive and were used only to characterize the sensitivity of the study, not to justify the adequacy of the sample size.

## 3. Results

### 3.1. Physical Performance

#### 3.1.1. Strength Performance

**Figure 2:**
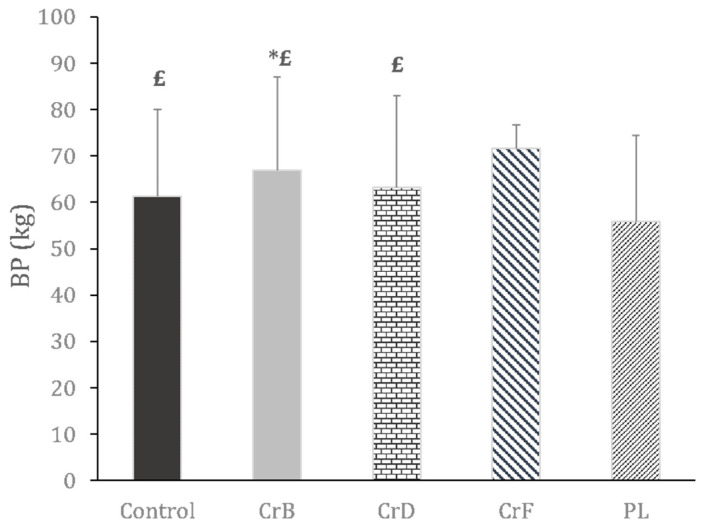
Physical performance during bench press (BP) recorded under each condition in physically active men (n = 11). Values are presented as mean ± SD. *: Higher performance than control; £: higher performance than placebo; CrB: creatine before; CrD: creatine during; CrF: creatine following; PL: placebo.

There was a significant effect of condition for BP performance (F_(1.35,13.54)_ = 4.91; ηp^2^ = 0.33; *p* = 0.035). The post hoc analysis revealed that CrB elicited a higher lifted weight than PL (95% CI_d_ = 2.97 to 18.85; d = 0.56; *p* = 0.006) and control (95% CI_d_ = 0.97 to 9.94; d = 0.28; *p* = 0.014). Moreover, values under CrD (95% CI_d_ = 1.18 to 13.36; d = 0.38; *p* = 0.016) and control (95% CI_d_ = 1.67 to 9.24; d = 0.29; *p* = 0.004) conditions were higher than those under PL (Figure 2).

**Figure 3:**
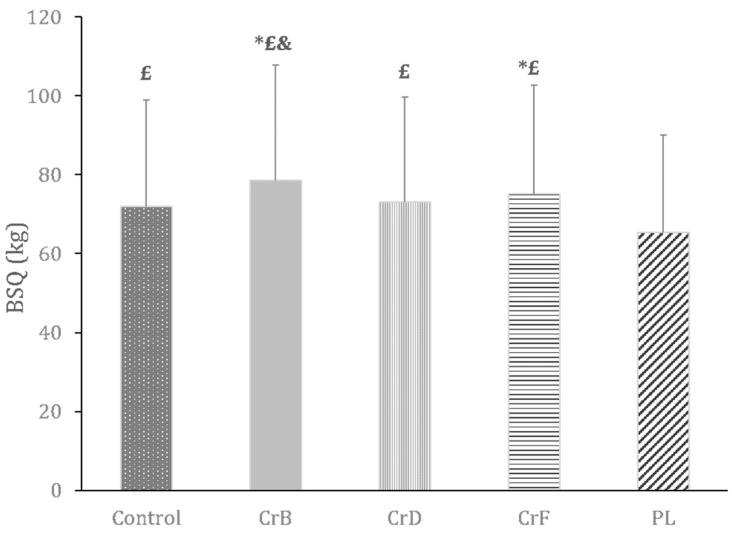
Physical performance during back squat (BSQ) recorded under each condition in physically active men (n = 11). Values are presented as mean ± SD. *: Higher performance than control; £: higher performance than placebo; &: higher than CrD; CrB: creatine before; CrD: creatine during; CrF: creatine following; PL: placebo.

For BSQ performance, there was a significant effect of condition (F_(1.91,19.06)_ = 33.22; ηp^2^ = 0.77; *p* < 0.001). The post hoc analysis revealed that CrB elicited higher values than CrD (95% CI_d_ = 0.57 to 10.53; d = 0.20; *p* = 0.026), PL (95% CI_d_ = 6.67 to 19.69; d = 0.49; *p* < 0.001), and control (95% CI_d_ = 3.69 to 9.56; d = 0.24; *p* < 0.001). Moreover, CrF elicited higher values than PL (95% CI_d_ = 4.59 to 14.86; d = 0.37; *p* < 0.001) and control (95% CI_d_ = 0.06 to 6.31; d = 0.12; *p* = 0.045). In addition, higher values were recorded under CrD (95% CI_d_ = 4.69 to 10.59; d = 0.30; *p* < 0.001) and control (95% CI_d_ = 2.61 to 10.48; d = 0.25; *p* = 0.001) conditions as compared to PL (Figure 3).

#### 3.1.2. CMJ Performance

There was no effect of condition on CMJ [power (χ^2^ (4) = 1.38; *p* = 0.85); height (χ^2^ (4) = 4.95; *p* = 0.29); strength (χ^2^ (4) = 2.94; *p* = 0.57); speed (χ^2^ (4) = 4.15; *p* = 0.39)] (Table 1).

### 3.2. Blood Variables

The Friedman test showed a significant effect of condition on CK (χ^2^ (4) = 12.22; *p* = 0.016). The Wilcoxon test showed that the CK values were lower under the control condition compared to the CrD condition (Z = −2.31; *p* = 0.021; r = −0.70) and CrF condition (Z = −2.49; *p* = 0.013; r = −0.75). Additionally, the CK concentrations were lower under the PL condition than those recorded under the CrF condition (Z = −2.04; *p* = 0.041; r = −0.62). Moreover, there was a significant effect of condition on creatinine (χ^2^ (4) = 17.75; *p* = 0.001). The Wilcoxon signed-rank test showed that creatinine concentrations were significantly lower in the CrB compared to the CrD (Z = −2.13; *p* = 0.033; r = −0.64), the CrF (Z = −2.934; *p* = 0.003; r = −0.88), and the control condition (Z = −2.31; *p* = 0.021; r = −0.70). Additionally, the PL condition elicited lower creatinine concentrations than the CrF condition (Z = −2.13; *p* = 0.033; r = −0.64). However, no significant effect of condition for the LDH concentrations (χ^2^ (4) = 3.34; *p* = 0.50), ALP concentrations (F_(4,7)_ = 3.89; η_p_^2^ = 0.69; *p* = 0.057), ALT (χ^2^ (4) = 6.65; *p* = 0.16), AST (χ^2^ (4) = 3.42; *p* = 0.49), K^+^ (F_(4,7)_ = 1.54; η_p_^2^ = 0.47; *p* = 0.29) or Na^+^ (F_(4,7)_ = 2.26; ηp^2^ = 0.56; *p* = 0.16) (Table 2).

### 3.3. Profile of Mood States

The Friedman test revealed that there was no significant effect of condition on fatigue–inertia (χ^2^ (4) = 6.84; *p* = 0.15), anger (χ^2^ (4) = 2.06; *p* = 0.73), vigor (F_(4,7)_ = 0.38; ηp^2^ = 0.18; *p* = 0.82), confusion (χ^2^ (4) = 2.89; *p* = 0.58), depression (χ^2^ (4) = 3.79; *p* = 0.44), or tension (χ^2^ (4) = 1.40; *p* = 0.84) (Table 3).

### 3.4. Digit Cancelation Test

There was no significant effect of condition on DCT scores (F_(4,7)_ = 3.98; ηp^2^ = 0.69; *p* = 0.054) (Table 4).

### 3.5. RPE, PRS, and DOMS

The Friedman test showed that there was no effect of condition on RPE (χ^2^ (4) = 9.08; *p* = 0.06), PRS (χ^2^ (4) = 3.64; *p* = 0.46) or DOMS scores (F_(4,7)_ = 1.31; ηp^2^ = 0.43; *p* = 0.35) (Table 4).

## 4. Discussion

This randomized crossover pilot study examined whether the timing of a single dose of Cr ingestion relative to a resistance training session influenced acute performance or physiological and perceptual responses in physically active men. The main finding was that Cr ingestion before exercise (CrB) significantly enhanced strength performance during both BP and BSQ compared with PL and no-supplement control conditions. Conversely, ingesting Cr during (CrD) or after exercise (CrF) conferred no such ergogenic benefits. Furthermore, Cr timing did not modify CMJ performance, cognitive function, POMS, RPE, PRS, or DOMS. Regarding circulating biomarkers, acute alterations were limited to greater CK concentrations in CrF and lower creatinine concentrations in CrB. Taken together, the data indicate that ingesting creatine before training may provide the most consistent acute strength benefit when no loading phase is used, with limited influence on neuromuscular power, cognitive outcomes, or subjective recovery. Practically, athletes using a single creatine dose may benefit from taking it about two hours before resistance training when the primary goal is to enhance acute strength performance.

While several investigations and reviews have highlighted the potential impact of Cr timing [4,8,32], a small number of original investigations have explored the potential efficacy of chronic timed Cr administration, displaying mixed outcomes [10,11,12]. Over multi-week training protocols, some evidence points to superior muscle mass and strength adaptations when Cr is consistently consumed post-exercise rather than pre-exercise [8,10,11]. By contrast, other evidence shows distinct strength improvements by distributing Cr throughout training Mills, Candow, Forbes, Neary, Ormsbee and Antonio [12]. While evidence regarding chronic effects of Cr intake around strength exercise relies on two-point comparisons, the present study is the first to address the acute effect of Cr ingestion around strength training sessions using three time points (before, during, after). Skeletal blood flow kinetics likely explain the distinct ergogenic advantage observed with pre-exercise delivery, When providing Cr before exercise, increased plasma Cr concentrations coincide with increased blood flow that occurs during the exercise [8]. Such an increase in blood flow kinetics and capillary perfusion during resistance exercise can result in greater delivery, retention, and metabolization of the nutrients to the exercised muscles [33]. This acute kinetic alignment supports the physiological model that pre-exercise ingestion maximizes muscle Cr availability more effectively than intra- or post-exercise protocols [8]. However, the final magnitude of this ergogenic response may remain bound to individual biological profiles [34]. In fact, evidence showed a person-by-treatment interaction to acute Cr supplementation, with one responder possessing a biological profile of the lowest baseline phosphocreatine (PCr) concentrations, greatest percentage of type II fibers, and greatest preload muscle fiber cross-sectional area [34]. These factors may modulate Cr ergogenic potential and explain, in part, the convergence across studies. Within this broader and heterogeneous body of literature, the present findings isolate the acute, single-dose timeline, demonstrating that immediate pre-exercise Cr delivery yields clear advantages for acute performance before chronic accumulation takes place.

Circulating biochemical markers exhibited modest changes in circulating CK and creatinine concentrations, whereas broader hepatic, metabolic, and electrolyte profiles remained entirely stable. The observed elevation of CK following post-exercise Cr ingestion likely reflects altered creatine–phosphocreatine turnover and accelerated CK enzyme activity during early recovery rather than exacerbated structural muscle damage [35]. Although Cr supplementation has often been proposed to attenuate muscle damage, previous studies have reported inconsistent effects on CK responses [36,37]. The magnitude of these biochemical fluctuations may be influenced by training status and exercise characteristics. Less-trained individuals appear to exhibit greater Cr uptake and larger responses to supplementation than trained individuals [38,39], limiting the generalizability of findings from untrained populations to resistance-trained participants. Overall, the results suggest that Cr timing may influence acute biochemical responses without clear implications for muscle damage, reinforcing the notion that pre-exercise Cr ingestion may be effective when the goal is to enhance acute strength performance rather than to modify recovery-related biomarkers.

Creatinine is a metabolic by-product derived from creatine and phosphocreatine turnover in skeletal muscle [40]. In the present study, creatinine was lower under the CrB condition compared to CrD, CrF, and the control condition. This finding is partially consistent with previous work suggesting that Cr supplementation and exercise can influence circulating creatinine concentration [13]. However, interpretations of these changes should be made with caution. Plasma creatinine reflects the balance between its production and renal excretion and is widely used as an indicator of renal function [2]. Importantly, post-exercise creatinine concentrations can be influenced by multiple factors, including hydration status, renal handling, exercise-induced stress, and timing of sampling, rather than reflecting a specific metabolic mechanism [41]. The higher creatinine concentrations observed in CrD and CrF should not be interpreted as direct evidence of altered ATP resynthesis. Although high-intensity exercise may contribute to increased creatinine through enhanced muscle turnover [42], the present data do not allow confirmation of the underlying mechanisms. As such, these findings should be interpreted as descriptive, and any mechanistic explanation remains speculative in the absence of direct physiological measurements.

It is well known that the daily dose and duration of Cr intake modulate the rate and intensity of Cr storage [43]. Regarding dose, the acute, single-dose design of this study introduces specific metabolic considerations regarding muscular saturation. Given that human daily Cr degradation rests at approximately 1.6% (~2 g) [44], our acute dose was, probably, sufficient to meet baseline maintenance demands. Yet, its capacity to induce immediate performance shifts may have been blunted if participants possessed high baseline intramuscular Cr stores [45]. This potential saturation effect likely explains the lack of observable changes in cognitive function and perceptual metrics. Furthermore, matching the timelines of comparable acute crossover designs, a 7-day washout period was used [28,29]. While this period represents a practical compromise to preserve training continuity and participant adherence [46], full normalization of intramuscular creatine stores may require four to six weeks following cessation [3,47]. Therefore, although the use of a single acute dose may reduce the likelihood of substantial creatine accumulation compared with chronic supplementation protocols, potential carryover effects between testing blocks cannot be entirely ruled out and warrant consideration. This issue should be considered when interpreting the present findings, and future crossover studies should consider longer washout periods or direct assessment of muscle creatine concentrations.

Considering the treatment duration, a common misconception about Cr supplementation is that a “loading phase” is essential to achieve the supposed ergogenic benefits [48]. In this respect, previous research has established the efficacy of combining Cr loading with resistance training [13,14] to accelerate augment adaptations. In the present study, one training session was used based on hypotheses that timing effects are most pronounced during the initial loading phase (i.e., first 2 days), and may be irrelevant once muscle channels saturate [43]. However, while the muscle Cr was not measured, it seems that a single acute dose of Cr without loading may not replicate chronic supplementation effects. This could be relevant since a high single dose of Cr (i.e., 0.35 g/kg) can reverse metabolic alterations and fatigue-related cognitive deterioration [28]. On the other hand, the substantial variability observed across several outcomes likely reduced the sensitivity to small but meaningful effects, thereby increasing the risk of a Type II error. Accordingly, non-significant findings, especially for secondary outcomes, should be interpreted with caution, as they may reflect limited sensitivity rather than a true absence of effect.

To the best of our knowledge, this is the first study to simultaneously map physical performance, cognitive function, and perceived recovery following an acute, single dose of timed Cr supplementation around a resistance training session. While the outcomes emphasize the unique value of pre-training delivery for acute strength performance, several limitations shape their generalizability. The findings provided by this experiment may confirm the need for a loading period to elicit significant improvement of muscle strength and power benefits [47]. Despite meeting a priori power requirements for primary strength outcomes (see Supplementary Material), this study represents a pilot trial characterized by a small cohort (n = 11) evaluated across five complex experimental conditions. Therefore, the findings should be interpreted with caution and should not be generalized without confirmation in larger samples. Although a placebo condition was included, it effectively controlled for placebo-induced effects only in the pre-exercise ingestion condition. In contrast, under the creatine-during and -following exercise conditions, participants were necessarily aware of the timing of supplementation, which may have confounded blinding and expectancy effects and influenced performance or perceptual responses [49]. Even though participants were asked to follow their habitual diet, the study’s findings are also limited by the absence of dietary control. Variability in athletes’ nutritional intake may have influenced baseline Cr store and metabolic responses, which should be considered when interpreting the results. Individuals with lower baseline stores typically show greater increases, whereas those near saturation exhibit attenuated responses [8]. This is particularly relevant in crossover designs, where fluctuations in creatine stores between periods may introduce additional variability and potential carryover effects [50]. From a mechanistic perspective, no direct measurements of neuromuscular activation, muscle fiber morphology or recruitment, muscle protein turnover, satellite cell activity, growth factor signaling, hormonal responses, oxidative stress, or inflammatory markers were performed, restricting insight into the physiological pathways underlying the observed effects. Future mechanistic studies should aim to determine whether timing-related changes in muscle Cr content are attributable to enhanced Cr uptake or increased intramuscular retention, potentially using tracer infusion and microdialysis techniques. Finally, further research comparing Cr ingestion during exercise with Cr consumed before and after training at different times of the day may help clarify whether observed effects are specific to the exercise window or reflect broader timing-dependent processes.

## 5. Conclusions

In conclusion, this randomized crossover pilot study suggests that, in physically active men, ingesting a single dose of creatine before a strength exercise session is associated with greater acute strength performance than ingesting creatine during or after exercise, placebo ingestion, or no supplementation. These effects are specific to strength-related outcomes, as creatine timing did not influence power performance, cognitive function, or perceptual measures of fatigue and recovery under the acute conditions examined. Nevertheless, the findings are exploratory and should be confirmed with a larger sample size. Moreover, given the lack of a fully matched placebo-control structure, the findings should be interpreted with caution because the observed timing effect may be confounded by blinding and expectancy bias.

Given the acute nature of the protocol and the use of a single creatine dose, the present findings should not be extrapolated to chronic supplementation practices, for which loading and maintenance protocols remain the evidence-based approach for maximizing broader performance and recovery adaptations. Future studies should examine whether these timing-related effects persist with repeated dosing and across different populations, including women, highly trained athletes, and less physically active individuals.

## Figures and Tables

**Table 1:** Physical performance recorded during the countermovement jump (CMJ) test under each condition in physically active men (n = 11). Values are presented as median (IQR).

		CrB	CrD	CrF	PL	Control	Statistics
CMJ	Height (cm)	30.20 ± 31.50	30.60 ± 15.50	30.60 ± 15.50	31 ± 11.20	35.04 ± 10.10	MED/IQR
	Power (W/kg)	47.50 ± 45.70	47.10 ± 26.60	46.40 ± 15.30	53.50 ± 13.10	48.80 ± 16.10	MED/IQR
	Force (N/kg)	25.10 ± 6.70	24.30 ± 6.10	24.40 ± 3.10	26.70 ± 7	24.80 ± 7.80	MED/IQR
	Speed (cm/s)	243 ± 111	249 ± 79	250 ± 43	267 ± 31	263 ± 38	MED/IQR

CMJ: Countermovement jump; MED: median; IQR: interquartile; CrB: creatine before; CrD: creatine during; CrF: creatine following; PL: placebo.

**Table 2:** The biochemical variables recorded under each condition in physically active men (n = 11). Values are presented as mean ± SD for normally distributed variables (†) or median (IQR) for non-normally distributed variables (#).

	CrB	CrD	CrF	PL	Control	Statistics
CK (U/L) #	384 ± 787	580 ± 351	433 ± 686	400 ± 585 ^£^	288 ± 387 *	MED/IQR
LDH (U/L) #	240 ± 201	234 ± 23	226 ± 24	232 ± 55	209 ± 80	MED/IQR
ALP (U/L) †	155 ± 100.64	199.09 ± 91.85	226.63 ± 79.38	190.09 ± 89.65	222.36 ± 64.79	M(SD)
ALT (U/L) #	28/19	30/17	28/15	26/11	27/6	MED/IQR
AST (U/L) #	27 ± 17	32 ± 10	31 ± 15	31 ± 13	35 ± 12	MED/IQR
K+ (mmol/L) †	4.19 ± 0.28	4.22 ± 0.34	4.25 ± 0.40	4.29 ± 0.43	3.93 ± 0.41	M(SD)
Na+ (mmol/L) †	138.62 ± 5.05	142.02 ± 5.54	141 ± 5.91	138.51 ± 4.08	138.27 ± 4.05	M(SD)
Creatinine (μmol/L) #	96.30 ± 32.70 ^&^	105.40 ± 33.60	111.70 ± 30.10	90 ± 42.40	105.80 ± 15.20 ^ß^	MED/IQR

†: Normally distributed variables; #: non-normally distributed variables; *: significantly different compared to CrD and CrF (*p* < 0.05); £: significantly different compared to PL (*p* < 0.05); &: significantly different compared to CrD, CrF and control (*p* < 0.05); ß: significantly different compared to CrF (*p* < 0.05); CK: creatine kinase; LDH: lactate dehydrogenase; ALP: alkaline phosphatase; ALT: alanine aminotransferase; AST: aspartate aminotransferase; M: mean; SD: standard deviation; MED: median; IQR: interquartile; CrB: creatine before; CrD: creatine during; CrF: creatine following; PL: placebo.

**Table 3:** The Profile of Mood States subscale scores recorded under each condition in physically active men (n = 11). Values are presented as mean ± SD for normally distributed variables (†) or median (IQR) for non-normally distributed variables (#).

	CrB	CrD	CrF	PL	Control	Statistics
Fatigue (a.u) #	6 ± 5	10 ± 10	7 ± 10	3 ± 6	6 ± 8	MED/IQR
Anger (a.u) #	9 ± 10	9 ± 5	6 ± 16	6 ± 15	7 ± 18	MED/IQR
Vigor (a.u) †	22.90 ± 6.30	20.18 ± 7.57	21.90 ± 6.18	21.18 ± 5.79	23.18 ± 4.75	M (SD)
Confusion (a.u) #	6 ± 4	8 ± 7	6 ± 5	5 ± 11	6 ± 11	MED/IQR
Depression (a.u) #	4 ± 12	3 ± 7	6 ± 13	4 ± 16	3 ± 21	MED/IQR
Tension (a.u) #	8 ± 8		8 ± 7	7 ± 12	7 ± 15	MED/IQR

†: Normally distributed variables; #: non-normally distributed variables; a.u: arbitrary unit; M: mean; SD: standard deviation; MED: median; IQR: interquartile; CrB: creatine before; CrD: creatine during; CrF: creatine following; PL: placebo.

**Table 4:** Cognitive performance, perceived exertion, perceived recovery and delayed-onset muscle soreness recorded under each condition in physically active men (n = 11). Values are presented as mean ± SD for normally distributed variables (†) or median (IQR) for non-normally distributed variables (#).

	CrB	CrD	CrF	PL	Control	Statistics
DCT (a.u) †	57.09 ± 10.74	58.36 ± 7.66	62.45 ± 9.36	63.45 ± 7.27	65.91 ± 5.80	M (SD)
RPE (a.u) #	4 ± 5	5 ± 2	3 ± 2	4 ± 2	3 ± 4	MED/IQR
PRS (a.u) #	6 ± 1	7 ± 4	6 ± 1	6 ± 3	7 ± 2	MED/IQR
DOMS (a.u) †	4.45 ± 1.57	4.54 ± 2.10	4.54 ± 2.65	5 ± 2.44	3.64 ± 2.20	M (SD)

†: Normally distributed variables; #: non-normally distributed variables; a.u: arbitrary unit; M: mean; SD: standard deviation; MED: median; IQR: interquartile; CrB: creatine before; CrD: creatine during; CrF: creatine following; PL: placebo; DCT: digit cancelation test; RPE: rate of perceived exertion; PRS: perceived recovery scale; DOMS: delayed-onset muscle soreness.

## Data Availability

The data presented in this study are available on request from the corresponding author due to ongoing analyses and the need to preserve the integrity of planned follow-up studies.

## Supplementary Materials

The following supporting information can be downloaded at: https://www.mdpi.com/article/10.3390/nu18111789/s1, Table S1: Consolidated Standards of Reporting Trials checklist. Table S2: Post hoc statistical power analysis of the study outcomes.

## Abbreviations

The following abbreviations are used in this manuscript:

Cr	Creatine
PL	Placebo
CrB	Creatine ingested before exercise
CrD	Creatine ingested during exercise
CrF	Creatine ingested following exercise
POMS	Profile of Mood States
1-RM	One-repetition maximum
RPE	Rating of perceived exertion
BP	Bench press
DCT	Digit cancelation test
BSQ	Back squat
PRS	Perceived recovery scale
DOMS	Delayed-onset muscle soreness
CK	Creatine kinase
LDH	Lactate dehydrogenase
AST	Aspartate aminotransferase
ALT	Alanine aminotransferase
ALP	Alkaline phosphatase
